# Phosphate restriction using a processed clay mineral reduces vascular pathologies and microalbuminuria in rats with chronic renal failure

**DOI:** 10.1186/s12882-022-02743-5

**Published:** 2022-04-28

**Authors:** Jacqueline Hofrichter, Kai Sempert, Claus Kerkhoff, Anne Breitrück, Reinhold Wasserkort, Steffen Mitzner

**Affiliations:** 1 Department of Extracorporeal Therapy Systems, Fraunhofer Institute for Cell Therapy and Immunology, Schillingallee 68, 18057 Rostock, Germany; 2 Rostock University Medical Center, Center for Internal Medicine, Nephrology Section, Schillingallee 35, 18057 Rostock, Germany; 3 Department of Biomedical Sciences, University of Osnabrück, Institute of Health Research and Education, Barbarastraße 22c, 49076 Osnabrück, Germany; 4 Present address: Queensland Brain Institute, University of Queensland, St. Lucia, 4071 Australia

**Keywords:** Chronic Kidney Disease, Chronic Renal Failure, Phosphate Binder, Clay Minerals, Montmorillonite-Illite Clay Minerals, 5/6 Nephrectomy, Microalbuminuria

## Abstract

**Background:**

The progression of chronic kidney disease (CKD) is associated with an increasing risk of cardiovascular morbidity and mortality due to elevated serum phosphate levels. Besides low phosphate diets and hemodialysis, oral phosphate binders are prescribed to treat hyperphosphatemia in CKD patients. This study reports on a processed clay mineral as a novel and efficient phosphate sorbent with comparable efficacy of a clinically approved phosphate binder.

**Methods:**

5/6 nephrectomized rats, which develop chronic renal failure (CRF), received a high phosphate and calcium diet supplemented with either a processed Montmorillonite-Illite clay mineral (pClM) or lanthanum carbonate (LaC) for 12 weeks. Levels of plasma uremic toxins, glomerular filtration rates and microalbuminuria were determined and the histomorphology of blood vessels and smooth muscle cells was analyzed.

**Results:**

5/6 nephrectomy induced an increase in plasma uremic toxins levels and progressive proteinuria. Treatment of CRF rats with pClM decreased observed vascular pathologies such as vascular fibrosis, especially in coronary vessels. The transition of vascular smooth muscle cells from a contractile to a secretory phenotype was delayed. Moreover, pClM administration resulted in decreased blood creatinine and urea levels, and increased glomerular filtration rates, reduced microalbuminuria and eventually the mortality rate in CRF rats.

**Conclusion:**

Our study reveals pClM as a potent phosphate binding agent with beneficial impacts on pathophysiological processes in an animal model of CKD. pClM effectively attenuates the progression of vascular damage and loss of renal function which are the most severe consequences of chronic renal failure.

**Supplementary Information:**

The online version contains supplementary material available at 10.1186/s12882-022-02743-5.

## Background

Chronic kidney disease (CKD) is defined by the progressive loss of renal function with a severe reduction of the glomerular filtration rate to <60 ml/min/1.73 m^2^ compared to >90 ml/min/1.73 m^2^ in healthy people [1]. Health implications due to CKD are complex, but elevated serum phosphate levels (>5.5 mg/dL) represent a major risk factor for the associated increased cardiovascular morbidity and mortality [2, 3]. Hyperphosphatemia raises calcium release from bones and induces an accelerated progressive vascular calcification and hypertrophy of arterial walls, which results in a mortality rate of approximately 50% in terminal CKD patients due to artery and cardiovascular diseases such as left ventricular hypertrophy (LVH), vascular remodeling, and myocardial fibrosis [4, 5]. Elevated uremic toxins such as phosphate and uric acid cause the transition of vascular smooth muscle cells (SMCs) from a contractile phenotype to an extracellular matrix (ECM) secreting phenotype that contributes to fibrotic cardiac remodeling and increased collagen deposition, both strongly associated with cardiorenal syndrome [6–8]. Pronounced microalbuminuria is often correlated with hypertension and is a well described prognostic marker for future cardiovascular events [9–11].

According to the clinical relevance of phosphate in CKD, dietary control of phosphate uptake is crucial throughout all stages of CKD, beginning from earliest stages up to dialysis-dependent end-stage renal disease [12]. The average oral ingestion is about 1.4 to 2.0 g phosphate per day and 40–60% of ingested phosphate is intestinally absorbed [13]. Because of the inability of intermittent dialysis to provide a continuously sufficient phosphate clearance, a low phosphate diet and the elimination of dietary phosphate by phosphate adsorbers within the gastrointestinal tract is considered to be mandatory [14]. Furthermore, some phosphate binders exert pleiotropic effects as they attenuate oxidative stress and inflammation, and reduce the circulating levels of uremic toxins [15]. Contrarily, although phosphate binders have certain intrinsic advantages, they also cause gastrointestinal side effects. Together with a high pill burden, those disadvantages are the main reason for the need of new low compliance-demanding phosphate binders with fewer side effects [16, 17].

This study proves the efficacy of a processed Montmorillonite-Illite clay mineral as a phosphate binding agent in uremia-exhibiting rats, which received a 5/6 nephrectomy, in comparison to lanthanum carbonate. These processed clay minerals provide a high phosphate binding capacity due to their specific four-layer structure, high ion exchange capacity, and high content of iron oxide [18]. Main target parameters of this study were (i) survival, (ii) reduction of kidney retention parameters, (iii) effects on vascular morphology, (iv) changes in cardiac muscle tissue, (v) effects on glomerular filtration rate and (vi) on microalbuminuria. This study indicates an efficient elimination of enhanced dietary phosphate, resulting in reduced vascular pathologies and reduced mortality in CRF rats. The exertion of several beneficial effects in the etiopathology of chronic renal failure reveals a processed Montmorillonite-Illite clay mineral as an interesting and valuable candidate for a new phosphate binding agent.

## Methods

### Phosphate adsorbers

Montmorillonite-Illite clay minerals were refined and provided by FIM Biotech GmbH (Berlin, Germany). Technical processing steps were elutriation, fine grinding, and calcination which resulted a in processed clay mineral (pClM). Ground lanthanum carbonate (LaC) tablets (Fosrenol® 750 mg, Shire Pharmaceuticals, Hampshire, Great Britain) were used as reference and positive control.

### Animal experiments

Male Wistar rats, weighing 200 to 220 g, received a stepwise 5/6 nephrectomy by removing one kidney and one week later 2/3 of the second kidney to induce chronic renal failure (*n* = 26), or sham surgery (*n* = 8) (Charles River Laboratories, Germany). Rats were allowed free access to rat chow and tap water during routine husbandry in a 12-h dark/light cycle at 21–22 °C. Untreated chronic renal failure (CRF, *n* = 12) and sham-operated rats (sham) received a high phosphate (disodium phosphate 1.2%, d/w) and calcium (1.2%, d/w) diet. Both nephrectomized treatment groups also received a phosphate-rich (disodium phosphate 1.2%, d/w) and calcium-rich (1.2%, d/w) diet in combination with either 2% (d/w) of a processed clay mineral (pClM, *n* = 6) or lanthanum carbonate (LaC, *n* = 8). Samples of urine and feces were collected using metabolism cages for 24 h fortnightly. After 12 weeks, rats were weighed and euthanized by an overdose of i.p. injected ketamine-xylazine (100/25 mg/kg BW) followed by retrobulbar blood collection and subsequent transcardial perfusion with PBS. Tissue samples were either drop-fixed in 4% paraformaldehyde or snap-frozen in liquid nitrogen.

All experimental procedures were approved by and conducted in accordance with the guidelines of the State Department of Agriculture, Food Security and Fisheries Mecklenburg-Western Pomerania (Section 6/Department 600, Protocol Number: TV 7221.3–1.1-005/13). The study was carried out in compliance with the ARRIVE guidelines. Rats were weighed weekly and health conditions were checked daily with regard to their fur (smooth), eyes (clean and open) and posture (normal). Animals that lost more than 20% of their body weight or showed abnormal behavior and signs of severe pain were excluded from the experiment.

### Phosphate measurement via ICP-OES

For the analysis of phosphate in feces of rats, phosphate was extracted according to the International Organization for Standardization DIN EN 16174 [19]. Total phosphate of solid samples was measured by inductively coupled plasma optical emission spectrometry (ICP-OES).

### Analytical chemistry and glomerular filtration rate (GFR)

Levels of uremic toxins in blood and urine were quantitatively determined using a wet-chemical colorimetric method (Cobas Mira Plus, Roche, Germany). Blood samples were centrifuged and plasma concentrations of urea, uric acid, and creatinine were determined. Phosphate concentration was determined in serum. The uremic retention solutes urea, uric acid, creatinine, and phosphate were measured in 24 h urine collection samples. The volume of excreted urine was documented for calculation of GFR and 24 h phosphate excretion and is shown as such. The GFR, creatinine clearance and blood urea nitrogen (BUN) clearance were calculated as values per animal according to S. Pestel et al. [20].

### Histochemistry and immunofluorescence

Paraffin-embedded tissues were cut into 4 μm thick histological sections using a microtome and subsequently deparaffinized and rehydrated before histochemical staining with hematoxylin and eosin dye (Medite, Burgdorf, Germany), or Masson’s Goldner trichrome (Sigma-Aldrich, Germany) according to the manufacturers protocol. For immunofluorescence labelling, antigen retrieval was performed after deparaffinization and rehydration in heated trisodium citrate buffer (pH 6), followed by incubation in blocking solution (4% BSA, 1x PBS, 0.1% Tween) for 1 h. The primary antibody against α-smooth muscle actin (α-SMA, Sigma-Aldrich, Munich, Germany) was incubated overnight and detected with Alexa Fluor® 594 anti-mouse IgG (Life Technologies, Ober-Olm, Germany). Fluorescence intensity and collagen content was imaged using a Nikon Eclipse Ti-E microscope (Nikon GmbH, Düsseldorf, Germany).

### Quantitative histological analyses

For quantification of α-SMA in aortic arch sections, five α-SMA positive areas were randomly selected and the average intensity was measured. Analyses of vascular fibrosis and ventricular hypertrophy from cross-sectional sections of paraffin-embedded hearts were performed according to the protocol described by Finch et al. with minor modifications [14]. In brief, after Masson’s Goldner trichrome staining (i) medial area to luminal area ratio and (ii) perivascular collagen area to luminal area ratio were determined to quantify the severity of cardiac fibrosis. Histological sections were analyzed using NIS-Elements AR software (Nikon GmbH, Düsseldorf, Germany).

### Western Blot

Total protein concentrations were determined using a BCA™ protein assay kit (Pierce, part of Thermo Fisher Scientific, Rockford, USA). Proteins of tissue homogenates were separated by SDS-PAGE using 20 μg total protein per lane. After blotting, membranes were blocked with 1% BSA and initially incubated overnight with primary antibody against α-SMA (1:1000). Blots were then stripped (24 mM glycine, 2% SDS, aqua dest, pH 2.0) at 65 °C, blocked with 1% BSA, and incubated overnight with primary antibody against GAPDH as a loading control (1:1500, Biomol, Hamburg, Germany). HRP-conjugated secondary antibodies (anti-mouse and anti-rabbit 1:10,000, GE Healthcare, Buckinghamshire, UK) were used as detection antibodies. Target proteins were visualized and quantified by Fusion Capt Advance FX7 detection software (Vilber Lourmat GmbH, Eberhardzell, Germany).

### Statistical analysis

Data are expressed as box plots with median (min to max). All data were tested for normal distribution using Kolmogorov-Smirnov normality test and analyzed by one-way ANOVA. P values *p* < 0.05 were considered as statistically significant. Levels of significance were determined as follows: *p < 0.05, **p < 0.01 and ***p < 0.001. All statistical calculations were performed using GraphPad Prism 6 (GraphPad Software Inc., San Diego, CA, USA).

## Results

### pClM treatment reduces serum phosphate levels

The binding capacity of pClM was thoroughly tested by conducting *in vitro* phosphate binding studies in comparison to LaC. Results revealed a comparable phosphate binding efficacy of pClM and LaC (see supplements Fig. S1A).

**Fig. 1:**
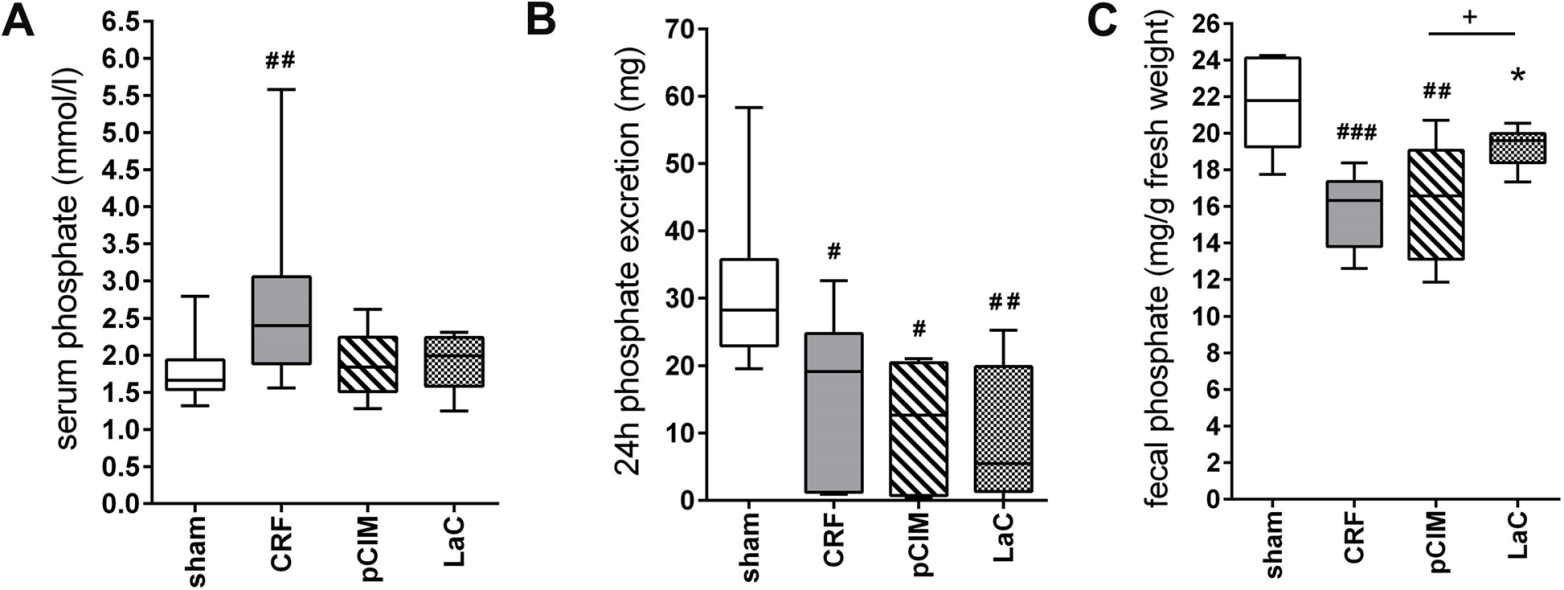
Phosphate levels of chronic renal failure rats (CRF) after 12 weeks of nephrectomy and phosphate and calcium enriched diet. **A** Serum phosphate was increased in untreated CRF rats whereas the serum phosphate level of phosphate adsorber treated CRF rats displayed control levels. **B** Excretion of phosphate during 24 h was decreased after 5/6 nephrectomy and even further after treatment with phosphate adsorbers. **C** Fecal phosphate concentrations were significantly decreased in nephrectomized rats compared to sham-operated rats. pClM treatment showed similar concentrations to untreated CRF rats. In contrast, LaC treatment resulted in significantly higher phosphate concentrations compared with untreated CRF and pClM-treated rats (min to max, ^#^vs. sham, *vs. CRF,  *p* ≤ 0.05; ^+^*p*ClM vs. LaC, *p* ≤ 0.05)

12 weeks after nephrectomy, serum phosphate levels were significantly increased by 52.0% in CRF rats compared to sham-operated controls, while levels in pClM- or LaC-treated animals were similar to sham-operated controls (Fig. 1A). Plasma concentrations of calcium, potassium and sodium showed no differences between the experimental groups (see supplements Fig. S4 B). However, compared to sham-operated rats 24 h phosphate excretion levels were significantly decreased by 50.1% in CRF rats and even further by 64.7% in pClM and 69.8% in LaC-treated animals (Fig. 1B). Stool sample analyses showed significantly decreased phosphate excretion in CRF rats and pClM-treated rats and similarly a strong trend after LaC treatment compared to the sham-operated animals. Despite the overall reduced fecal phosphate levels compared to sham-operated animals, LaC treatment led to significantly higher values than pClM treatment (Fig. 1C).

### pClM treatment prevents CRF induced vascular pathologies

**Fig. 2:**
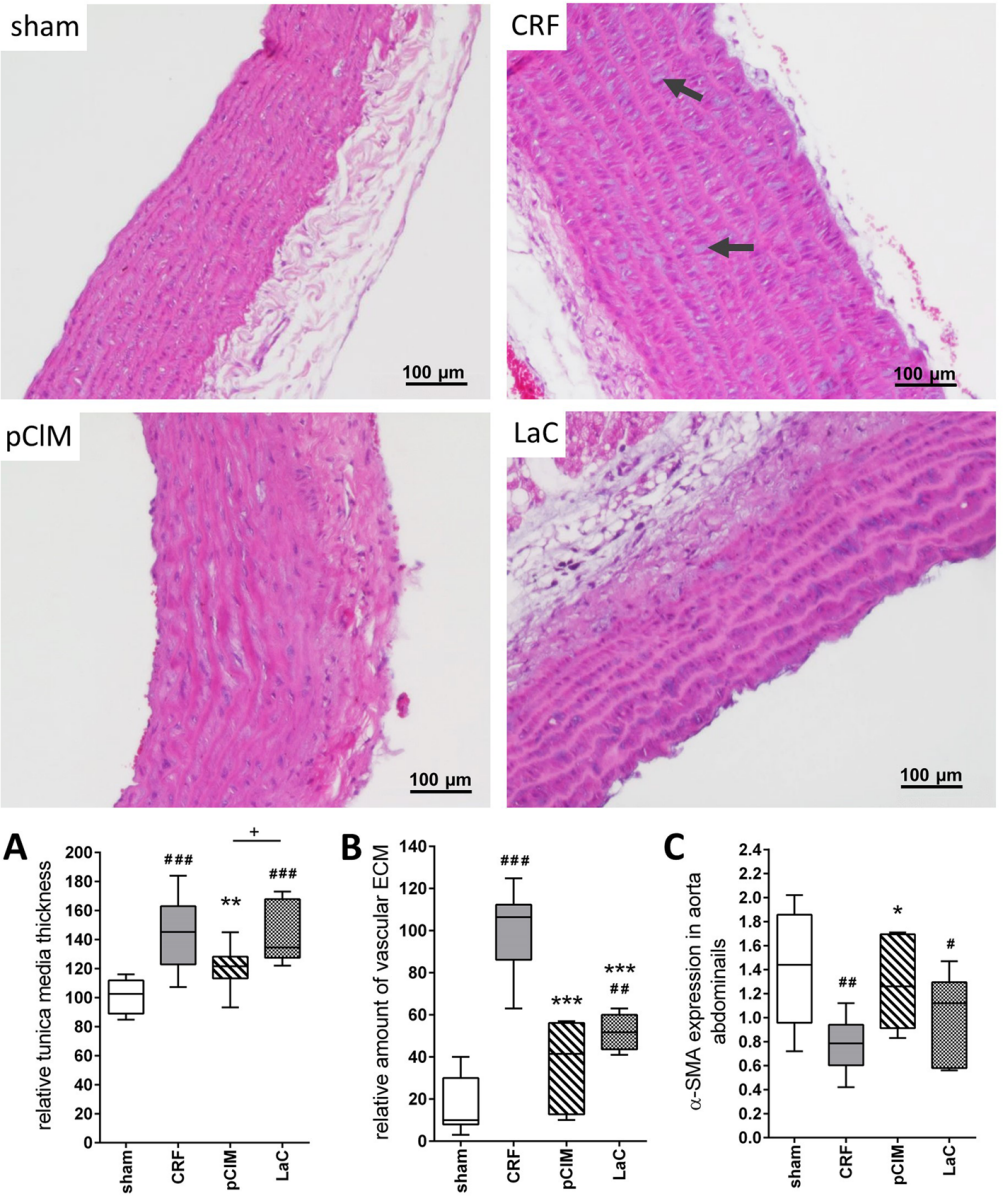
Effects of pClM and LaC on vascular pathologies in aortas of chronic renal failure rats (CRF). Hematoxylin and eosin staining was used to visualize the extracellular matrix (ECM) of abdominal aorta slices. **A** Treatment with a processed clay mineral (pClM) significantly prevented thickening of the vascular walls of the abdominal aorta and **B** synthesis of extracellular matrix (black arrows) within the tunica media. **C** Western blot analyses confirmed a reduction of α-SMA in the abdominal aorta of untreated CRF rats. Unlike LaC, pClM treated animals showed similar α-SMA expression levels as sham operated rats (min to max, #vs. sham, *vs. CRF, *p* ≤ 0.05)

Histological analyses of aortas from CRF rats revealed early arteriosclerotic stages such as significant thickening of the tunicae mediae which was prevented by pClM treatment but not by LaC (Fig. 2A). Furthermore, 5.5-fold increased formation of extracellular matrix (ECM) within interstitial spaces due to nephrectomy was significantly attenuated by pClM and to a markedly lesser degree by LaC (Fig. 2B). Western blot analyses showed that α-SMA expression levels were significantly decreased in CRF rats compared to sham-operated rats, which corresponds with an increased production of ECM (Fig. 2C). Remarkably, the pClM-treated CRF rats exhibited a stronger α-SMA expression compared to LaC-treated animals (Fig. 2C). These results were also confirmed by immunofluorescence-labelling against α-SMA. CRF rats showed reduced α-SMA immunofluorescence levels, which corresponded with increased production of ECM and enlarged interstitial spaces. Treatment with pClM prevented these alterations (see supplements Fig. S2, S3).

### pClM treatment prevents CRF-induced left ventricular hypertrophy and perivascular fibrosis

**Fig. 3:**
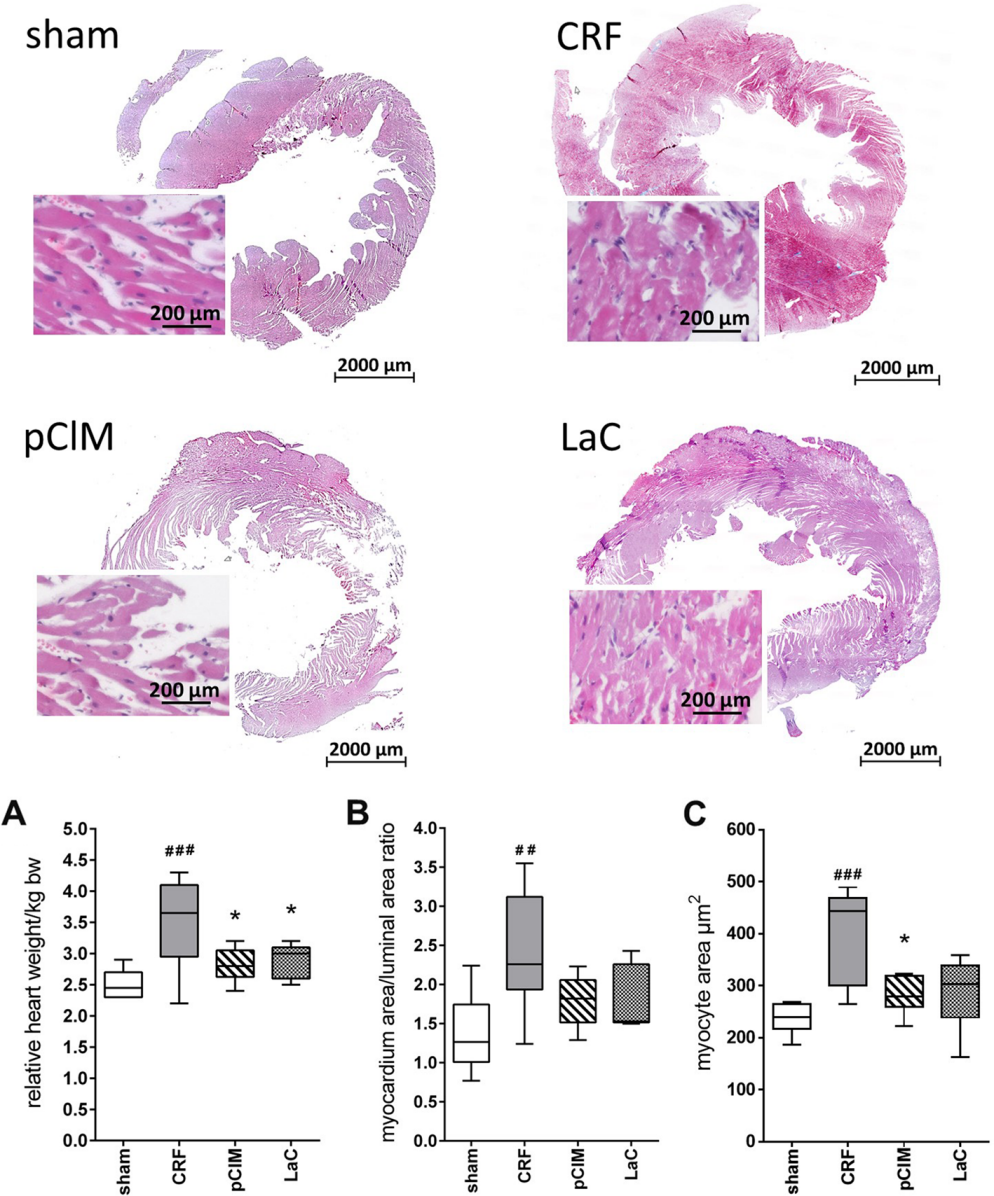
pClM prevents left ventricular hypertrophy caused by 5/6 nephrectomy and a high phosphate diet. **A** Measurement of heart weights indicates significantly hypertrophied hearts in untreated chronic renal failure rats (CRF) in contrast to the sham, pCIM and LaC groups. **B** The significantly enhanced myocardium/luminal area ratio in CRF rats implies a left ventricular hypertrophy compared to sham operated rats. Treatment with pClM prevented this pathological development. **C** Quantification of the myocyte area showed a significant increase in CRF rats (min to max, #vs. sham, *vs. CRF, *p* ≤ 0.05)

Dissected hearts of experimental animals were weighed and the heart-to-body-weight ratio was calculated. This ratio revealed a heart enlargement of 40% within the CRF group (Fig. 3A). Notably, pClM and LaC prevented this pathological alteration. In fact, the hearts of rats treated with pClM or LaC were comparable in weight to those of sham-operated animals. The observed increase in the myocardium/luminal area ratio in histological sections of CRF rats indicates a hypertrophy of the left ventricle, which was not seen in sham operated rats. This phenotype was entirely prevented by pClM and LaC (Fig. 3B). Moreover, analyses of myocyte areas in CRF rats depict strongly enlarged myocytes. pClM treatment attenuated myocyte enlargement significantly compared to CRF rats which was not apparent after LaC treatment (Fig. 3C).

**Fig. 4:**
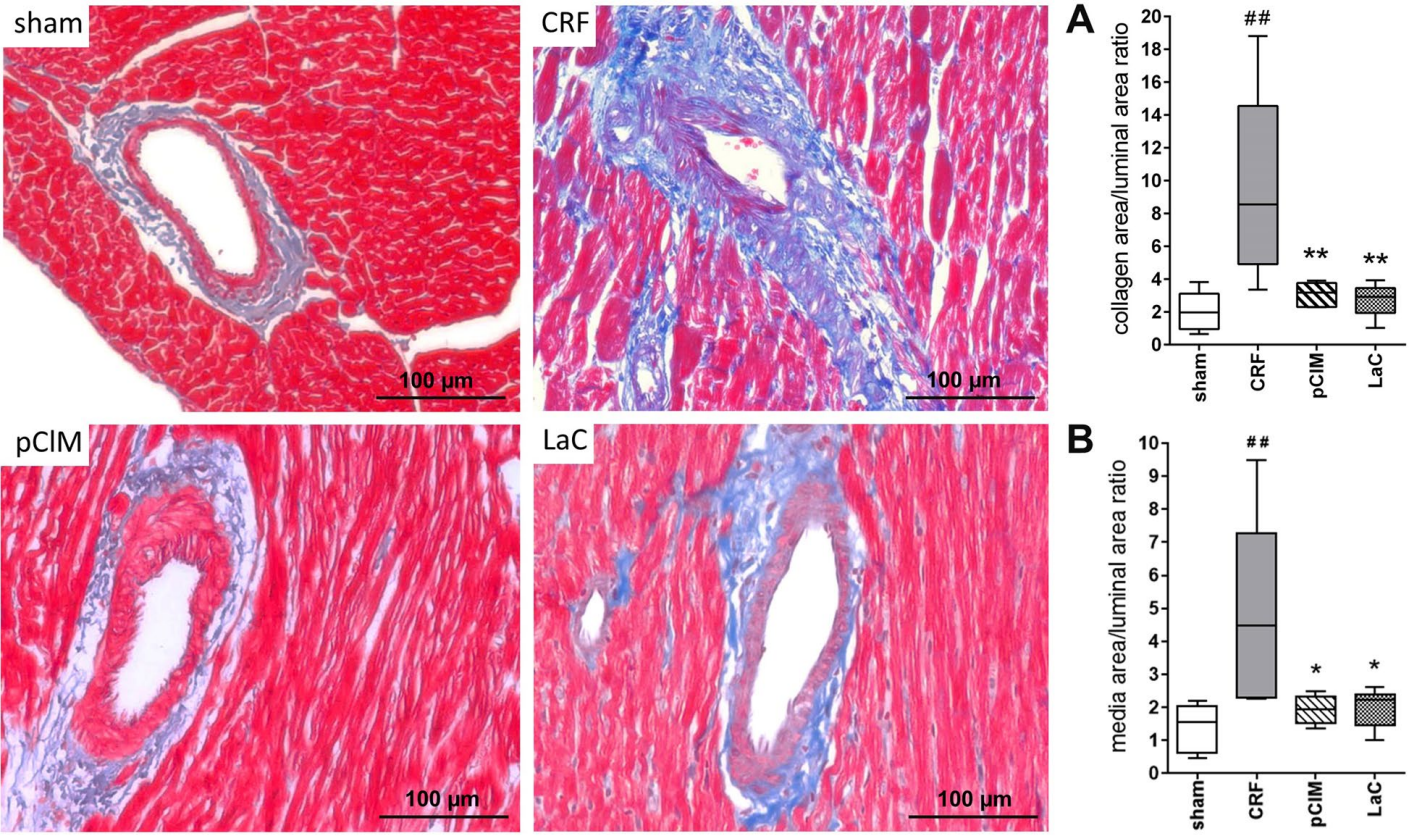
Reduction of fibrosis in coronary arteries of chronic renal failure rats (CRF) by pClM and LaC. **A** The perivascular collagen area (blue) to luminal area ratio of coronary arteries stained with Masson’s Goldner trichrome showed a perivascular fibrosis as consequence of 5/6 nephrectomy and a phosphate rich diet. Treatment with phosphate adsorber significantly prevented CRF rats from cardiovascular fibrosis. **B** The medial area to luminal area ratio indicates that adsorber treatment also prevented a swelling of the tunica media in coronary arteries (min to max, ^#^vs. sham, *vs. CRF, *p* ≤ 0.05)

Perivascular fibrosis was analyzed as the ratio of collagen surrounding the coronary vessels to the total perivascular area (Fig. 4). Perivascular fibrosis was dramatically increased in CRF and was prevented in rats treated with either pClM or LaC (Fig. 4A). Furthermore, the tunica media of coronary vasculature of CRF rats was significantly enlarged. The medial to luminal area ratio showed specifically a significantly enhanced thickening of the mediae from CRF rats and contrarily similarly lower values in sham, pClM, and LaC animals (Fig. 4B).

### pClM treatment maintains renal function

**Fig. 5:**
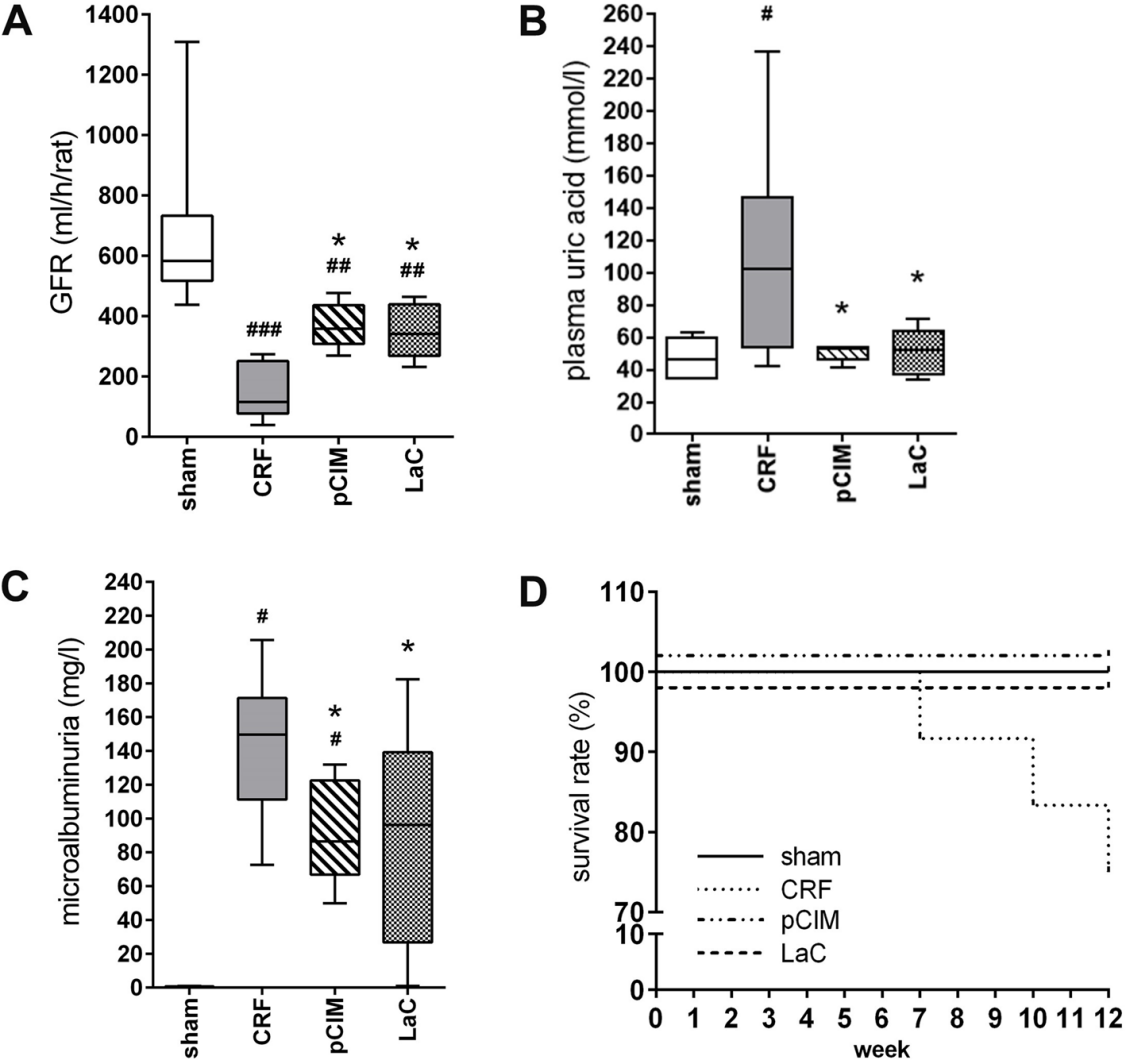
Preservation of renal function in chronic renal failure rats (CRF) rats by pClM and LaC. **A** The processed clay mineral (pClM) and lanthanum carbonate (LaC) partially attenuated the progressing reduction of the GFR in nephrectomized rats. **B** The increase of uric acid in plasma due to 5/6 nephrectomy was significantly decreased by pClM and LaC treatment comparable to sham operated levels. **C** pClM significantly prevented microalbuminuria in CRF rats. **D** Phosphate and calcium enriched diet raised the mortality rates by 25% in untreated CRF rats. In contrast, all sham operated rats and rats which received pClM or LaC survived the investigation period until dissection after 12 weeks (min to max, ^#^vs. sham, *vs. CRF, *p* ≤ 0.05)

A 5/6 nephrectomy resulted in a significantly reduced GFR compared to sham-operated controls (Fig. 5A). Treatment with both phosphate adsorbers attenuated this decrease in GFR compared to CRF rats. The increased levels of uremic toxins such as uric acid in plasma mirrors the decline in renal function. Untreated CRF rats exhibited 2-fold higher plasma uric acid levels compared to both treatment groups (Fig. 5B). Furthermore, microalbuminuria was strongly increased in all CRF rats but significantly reduced to sham levels only by pClM treatment (Fig. 5C). Importantly, untreated CRF rats showed a mortality rate of 25% while no death occurred in the pClM or LaC groups (Fig. 5D). Also, all sham-operated rats survived until dissection.

## Discussion

A balanced phosphate metabolism is essential to maintain health and well-being. Hyperphosphatemia due to reduced phosphate excretion during renal failure in chronic kidney disease (CKD) results in several related complications such as increased vascular calcification and, therefore, enhanced cardiovascular risk [21, 22]. The inefficient ability of dialysis to remediate elevated blood phosphate concentrations to physiological levels, raises the need to reduce the phosphate uptake to a minimum [23, 24]. This is realized by a low phosphate diet supplemented with phosphate adsorbers.

In this study, we present evidence that a specifically processed Montmorillonite-Illite clay mineral (pClM) efficiently binds phosphate and has increased beneficial effects on cardiovascular risk factors in 5/6 nephrectomized Wistar rats in comparison to lanthanum carbonate (LaC), a clinically used phosphate adsorber. The special four-layer structure and the small particle size as well as their mineralogical and chemical composition facilitate the strong ion-exchange capacity of clay minerals (ClM) [18]. If specifically processed to pCIM, ClM possesses a higher phosphate binding capacity compared to LaC (see supplements Fig. S1A). The inability of aqua regia and microwave digestion to sequester the bound phosphate from pClM suggests a strong chemical stability once the binding process is completed (see supplements Fig. S1B). Based on these results, pClM was the only ClM used for further animal experiments in this study.

5/6 nephrectomy in rats resulted in significantly increased plasma phosphate levels whereas both, pClM and LaC, successfully prevented this phenotype (Fig. 1A). Additionally, we found that the 24 h phosphate excretion rate was reduced after 5/6 nephrectomy in rats and even further by treatment with pClM and LaC. This indicates a decreased phosphate uptake by the intestines due to the phosphate binding properties of the adsorbers during the gastrointestinal passage. Additionally, LaC rats showed increased fecal phosphate excretion compared to CRF rats and interestingly also to pClM rats. This is probably due to the fact that the binding between phosphate and pClM is extremely stable as demonstrated by the inability of aqua regia to removed more than 5% of the bound phosphate. In contrast, this method almost completely removed the phosphate bound to lanthanum carbonate.

Consequences of hyperphosphatemia are vascular pathologies e.g. the production of ECM which enhances the resistance of blood vessels and subsequently induces hypertension [25–27]. In this study, highly elevated formation of ECM was observed in 5/6 nephrectomized rats compared to sham-operated rats (Fig. 2 B). While in pClM-treated animals the amount of ECM was almost reduced to sham levels, LaC-treated animals still revealed a significantly higher quantity than the sham group. The tunica media thickness of the abdominal aorta exhibited a similar pattern (Fig. 2A). Interestingly, only treatment with pClM but not LaC was capable to reduce this pathological alteration. Consistent with these results, the relative amount of α-SMA within vascular smooth muscle cells (VSMC) was significantly lower in CRF rats as shown by Western Blot analyses (Fig. 2C). The loss of α-SMA suggests a phenotypic transition from contractile to secretory cells, as shown by Wang et al. [8, 28]. This is conclusively consistent with increased ECM production as shown for CRF rats. Only pCIM prevented this transition completely whereas LaC showed an incomplete prevention. However, hypertrophy of the whole heart and the left ventricle in particular as well as the increased myocyte size developed after 5/6 nephrectomy were avoided by treatment with pClM or LaC (Figs. 3 and 4). Thus, pClM effectively attenuates the progression of vascular injury which is the most severe consequence of chronic renal failure.

Various studies revealed a pronounced proteinuria as a reliable marker for future cardiovascular events such as stroke in CKD patients [29, 30]. In line with these studies, untreated 5/6 nephrectomy rats displayed a microalbuminuria, which was attenuated by pClM treatment (Fig. 5C). Interestingly, excessive urinary microalbumin concentrations could be observed in those animals (all from the group of untreated CRF rats) that died before the intended sacrifice (Fig. 5D). Likewise, arterial hypertension could have contributed to both: left ventricular hypertrophy and microalbuminuria. In this study, blood pressure measurements could not be conducted. Nevertheless, the phosphate-rich diet and subsequent vascular pathologies might have contributed to potentially increased blood pressure levels. It should also be pointed out that the demonstration of potent phosphate binding in an animal model of CKD may not directly translate to similarly strong effects in humans. Nevertheless, based on our findings, further examinations are indicated due to the promising results in this study. The demonstrated higher binding efficacyi combined with the improved ability of pClM to reduce several cardiovascular risks compared to the clinically used phosphate adsorber LaC strongly suggest to proceed the investigation of pClM to elucidate its full potential.

## Conclusions

In summary, pClM and LaC revealed beneficial effects in regard to the reduction of phosphate levels in blood and urine, retention parameters, and attenuating the decline of the GFR in CRF rats. However, pClM exhibited a stronger potential to counteract the development of cardiovascular pathologies. Remarkably, pClM was able to attenuate the decrease in GFR due to 5/6 nephrectomy, to prevent the transition of contractile VSMCs to cells with a secretory phenotype, and left ventricular hypertrophy. These results, especially the reduced excretion of microalbumin, suggest that the processed Montmorillonite-Illite clay mineral bears the potential as a highly potent phosphate binder to treat CKD-associated cardiovascular diseases, which makes it an intriguing candidate for further investigations.

## Supplementary Information

**Additional file 1.**

## Data Availability

The datasets generated during and/or analyzed during the current study are available from the corresponding author on reasonable request.

## Abbreviations

α-SMA: α-smooth muscle actin
CKD: Chronic kidney disease
ClM: Clay minerals
CRF: Chronic renal failure
ECM: Extracellular matrix
GFR: Glomerular filtration rate
ICP-OES: Inductively coupled plasma optical emission spectrometry
LaC: Lanthanum carbonate
LVH: Left ventricular hypertrophy
pClM: Processed clay mineral
SMC: Vascular smooth muscle cells
