# Supplementary material for: Phosphate restriction using a processed clay mineral reduces vascular pathologies and microalbuminuria in rats with chronic renal failure
Source: BMC Nephrol. 2022 Apr 28;23:162. doi: 10.1186/s12882-022-02743-5 (PMC9052552; doi:10.1186/s12882-022-02743-5)
Supplement: Supplementary file 1 — Additional file 1. [file 12882_2022_2743_MOESM1_ESM.zip › 222002_Supplements_Processed clay mineral in 5-6 NX rats_Revision2_v2.docx]

**Phosphate restriction with processed clay mineral reduces vascular pathologies and microalbuminuria in rats with chronic renal failure.**

# Phosphate binding studies

**1.1 Methods**

### **Phosphate binding capcaity of phosphate adsorbers**

Clay minerals (ClM) were processed to pCIM and provided by FIM Biotech GmbH (Berlin, Germany). Ground lanthanum carbonate (LaC) tablets (Fosrenol® 750 mg, Shire Pharmaceuticals, Hampshire, Great Britain) were used as positive control. Phosphate binding capacities of ClM and pClM were determined in ascending phosphate (Na_2_HPO_4_) concentration series in comparison to lanthanum carbonate: 25 mg adsorber was incubated under constant stirring in 1 ml phosphate solution of different concentrations (3 mM, 5 mM, 10 mM and 30 mM) at pH 5.0 for 12 hours.

### **Phosphate binding strength measurement via ICP-OES**

To determine the phosphate binding strength of the different phosphate adsorbers, either unprocessed clay minerals (CIM), a processed clay mineral (pCIM) or lanthanum carbonate (LaC) were incubated in a defined phosphate concentration (0.8 g adsorber with 2 ml 50 mM Na_2_HPO_4_, Carl Roth, Karlsruhe, Germany) for 14 h. Unbound phosphate in the supernatant was determined using Cobas Mira Plus (Roche, Mannheim, Germany) method. Afterwards, adsorber samples were washed three times with distilled water to thoroughly remove any unbound phosphate and then digested using aqua regia extraction to dissolve bound phosphate from the adsorber. The resolved phosphate residues were measured using Inductively Coupled Plasma Optical Emission Spectroscopy (ICP-OES).

## 1.2 Results

In ascending phosphate concentration series, the phosphate binding capacity of clay minerals prior (ClM) and after processing (pClM) was determined. Lanthanum carbonate (LaC) was used as a reference. Adsorber’s phosphate binding capacities increased according to ascending concentration of inorganic phosphate (Pi). The initially low binding property of CIM remained significantly lower compared to pCIM and LaC for all tested concentrations. Interestingly, whereas CIM and pCIM showed a linear increase of adsorbed phosphate in relation to the increasing phosphate concentration in solution, the values for LaC displayed a non-linear slope with a saturation effect obvious at 30 µM. At this concentration the amount of bound phosphate by pCIM was 44.5% higher compared to LaC (Fig S1A). The phosphate binding strength of the adsorbers were examined using aqua regia and microwave digestion followed by ICP-OES. The subsequently detected amounts of released phosphate were normalized to the difference of the total phosphate concentration in solution before and after incubation with the adsorbers to calculate the proportion of released phosphate to bound phosphate. Surprisingly, this procedure removed only a fraction of 4.8% bound phosphate from pClM. In contrast, 100% of bound phosphate were released from LaC. In regard to the overall lower phosphate binding capacity of LaC at higher phosphate concentrations (>30 µmol), this indicates a tremendously higher phosphate binding strength of pCIM compared to LaC (Fig S1B).


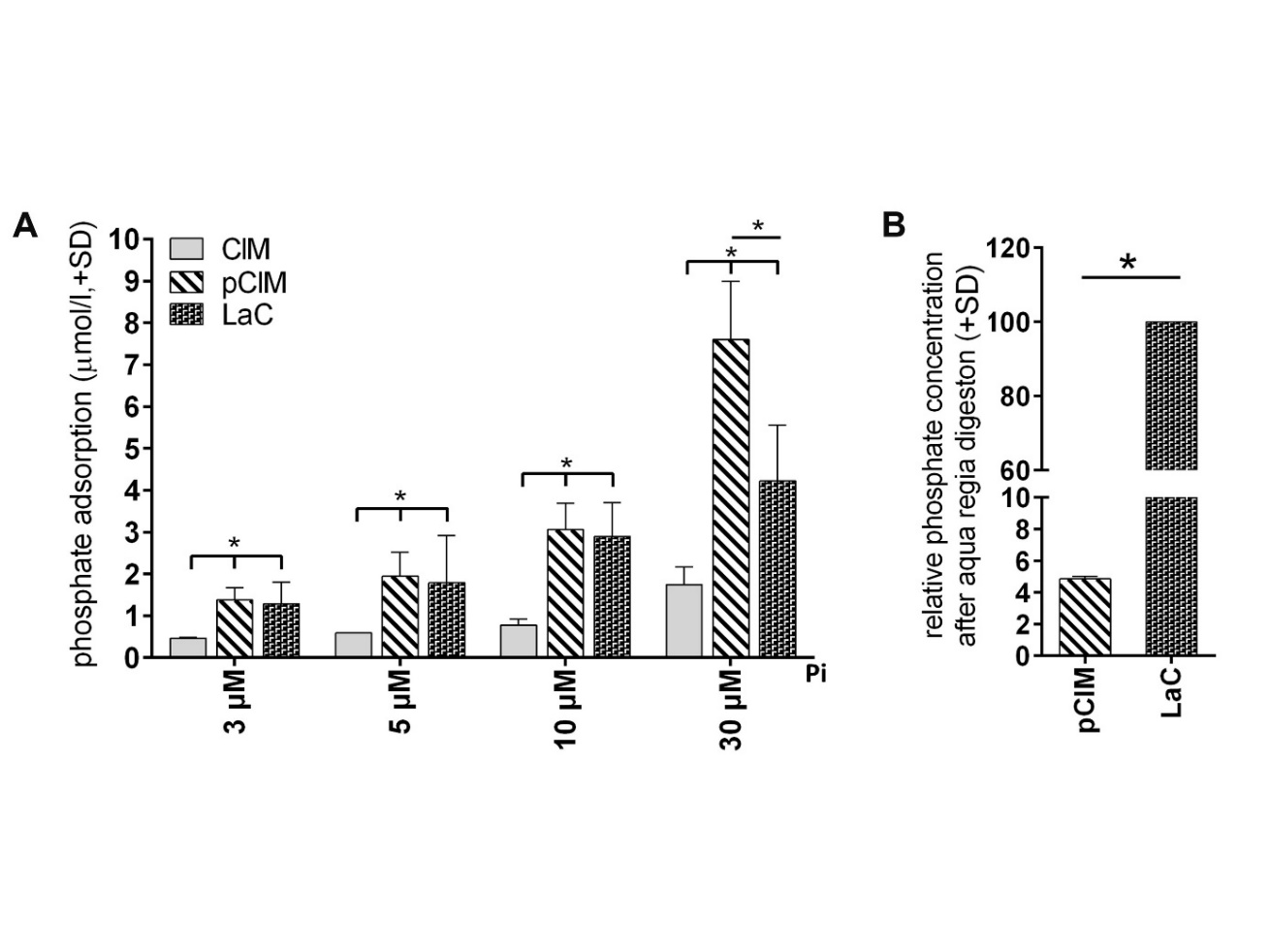


**Fig S1.** (A) *In vitro* phosphate binding studies showed an enhanced phosphate binding capacity of a processed clay mineral (pClM) compared to an unprocessed clay mineral (ClM). The values of phosphate binding between pClM and lanthanum carbonate (LaC) were comparable in efficiency at low phosphate concentrations. However, at 30 µM, the capacity of pClM was 44.5% higher than the capacity of LaC. (B) Aqua regia and microwave digestion of phosphate loaded adsorbers completely resolved phosphate from LaC, whereas only 4.8% of phosphate was removed from pClM (+SEM, *p≤0.05).

# α-SMA Immunohistochemistry

**2.1 Methods**

# Histochemistry and immunofluorescence

Refer to chapter ‘Methods’, section ‘Histochemistry and immunofluorescence’.

**2.2 Results**

5/6 nephrectomy in rats led to enlarged interstitial spaces in the aortic arches. The decreased expression of α-Smooth Muscle Actin (α-SMA) as shown in the representative images of α-SMA immunolabelling in aortic arches, and the corresponding graph (Fig S2A) indicates the depletion of the characteristic contractile phenotype of vascular smooth muscle cells. Both phosphate adsorbers (pClM, LaC) prevented this pathological alteration effectively.


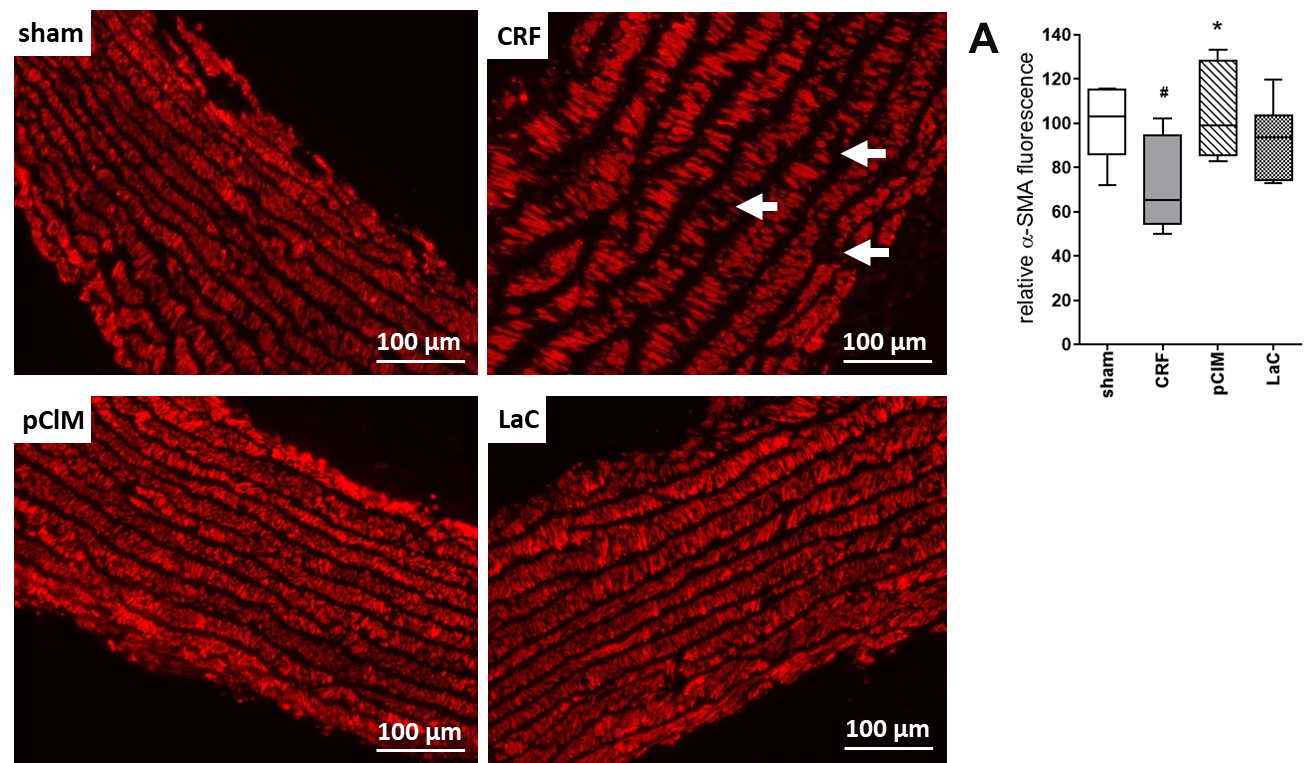


**Fig S2.** Preservation of a contractile phenotype of vascular smooth muscle cells. α-SMA immunofluorescence labelling of aortic arches showed enlarged interstitial spaces (white arrows) in rats with untreated chronic renal failure (CRF) and decreased α-SMA expression (A). This was prevented by processed clay mineral treatment (pClM) (min to max, #vs. sham, *vs. CRF, p≤0.05).

**
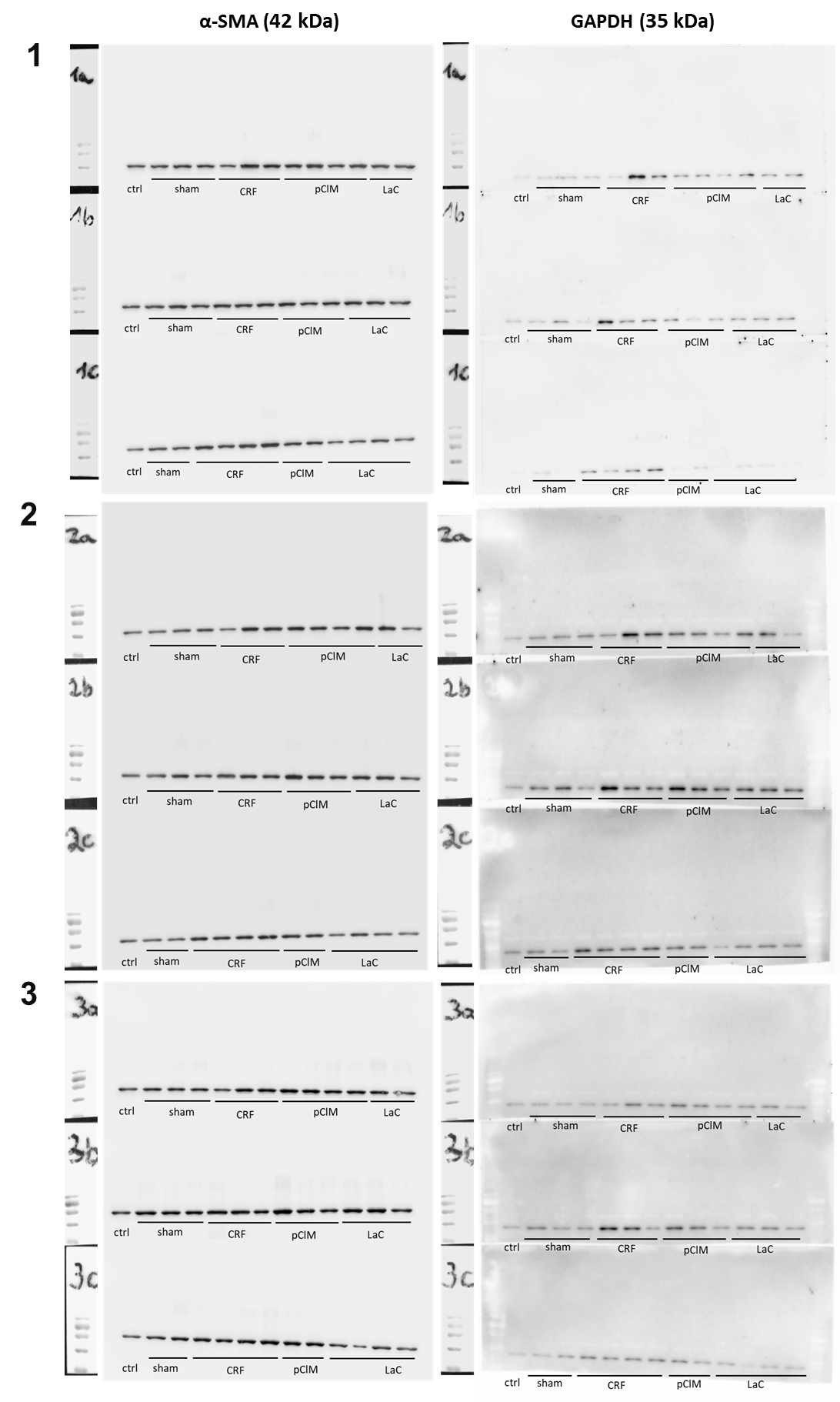
**

**Fig S3.** Western blots for densiometric analysis of α-SMA expression levels in the abdominal aorta of rats treated with a processed clay mineral (pClM) or lanthanum carbonate (LaC) compared to rats with untreated chronic renal failure (CRF) and sham operated rats (sham). (Left) Western blots for α-SMA (42 kDa) (technical replicates 1-3). (Right) Western blots for GAPDH (35 kDa) (technical replicates 1-3). A specific control sample (ctrl), was used as an internal control of blot quality on each blot. Live ladder recordings are shown to the left of the blots.

# In vitro and in vivo cation binding study for pClM

**3.1 Methods**

Binding capacities of calcium, sodium, potassium and magnesium by pClM were determined *in vitro*. In detail, 25 mg adsorber was incubated under constant stirring in 1 ml physiological solution containing calcium, sodium, potassium and magnesium (1,3 mmol/l; 138,0 mmol/l; 4,0 mmol/l; 0,8 mmol/l) at pH 5.0 for 12 hours. Cation concentrations (Ca^2+^, Na^+^, K^+^) were then determined by a blood gas analyzer (ABL90*flex*, Radiometer Medical ApS, Denmark). Magnesium concentration was determined using the Cobas Mira Plus (Roche, Mannheim, Germany) method.

Plasma cation levels of the rats used in this study were quantitatively determined using a blood gas analyzer (ABL90*flex*, Radiometer Medical ApS, Denmark) for potassium, calcium and sodium and Cobas Mira Plus method for determining magnesium concentrations.

**3.2 Results**

The *in vitro* binding studies confirm that the phosphate binder pClM does not bind calcium, potassium, sodium or magnesium. On the contrary, a significant release of these cations was observed during the binding experiments. These results were not unexpected as the analyzed cations are natural components in smectites of marine genesis (Fig. S4 A).

Importantly, no differences were detected in the plasma cation concentrations in the different experimental groups (Fig. S4 B) indicating that the observed release is not of physiological relevance in regard to CKD.


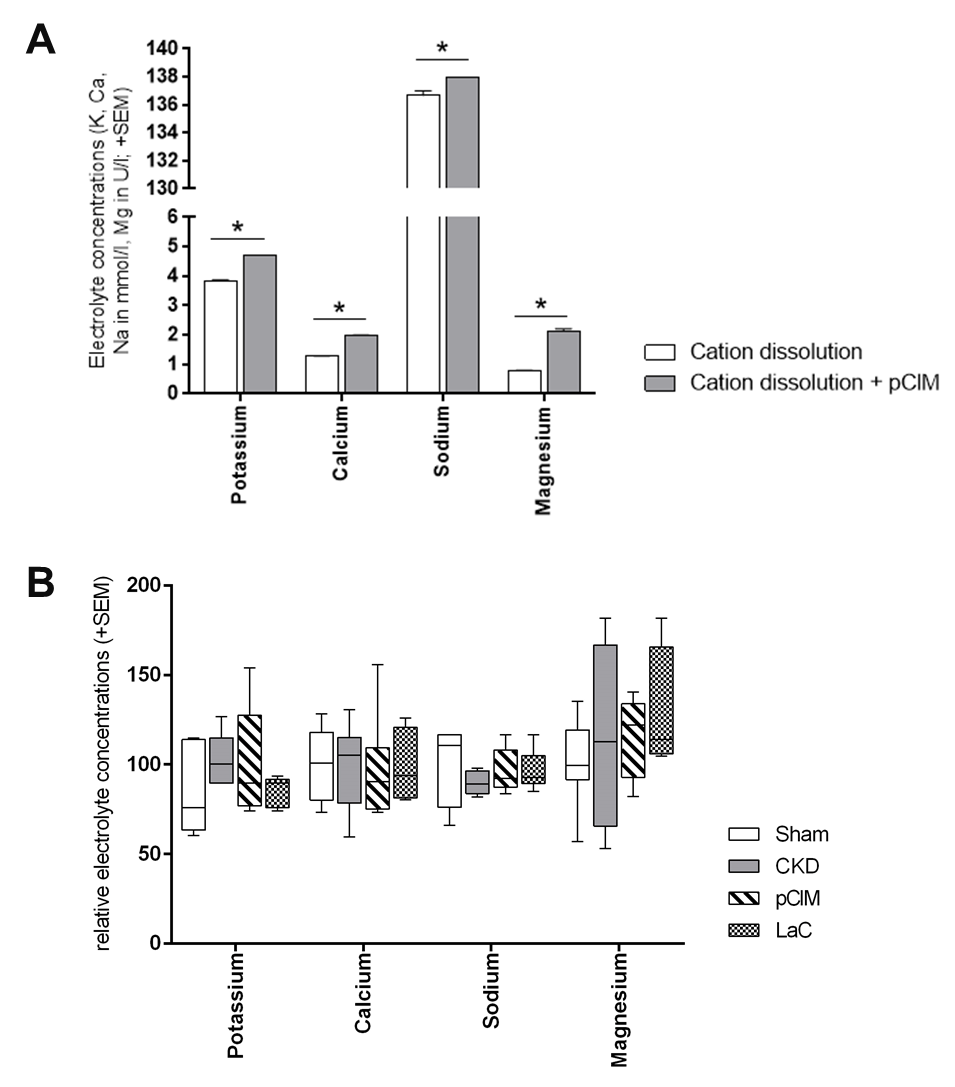


**Fig S4.** (A) *In vitro* cation binding studies revealed no binding of sodium, potassium, calcium, and magnesium by pClM. The study showed, on the contrary, a significantly increased cation release by pClM (n=3 consisting of triplicates, *p≤0.05). (B) Plasma concentrations of electrolytes showed no significant differences between all experimental groups (min to max).
