# Supplementary figures and images for: Phosphate restriction using a processed clay mineral reduces vascular pathologies and microalbuminuria in rats with chronic renal failure
Source: BMC Nephrol. 2022 Apr 28;23:162. doi: 10.1186/s12882-022-02743-5 (PMC9052552; doi:10.1186/s12882-022-02743-5)

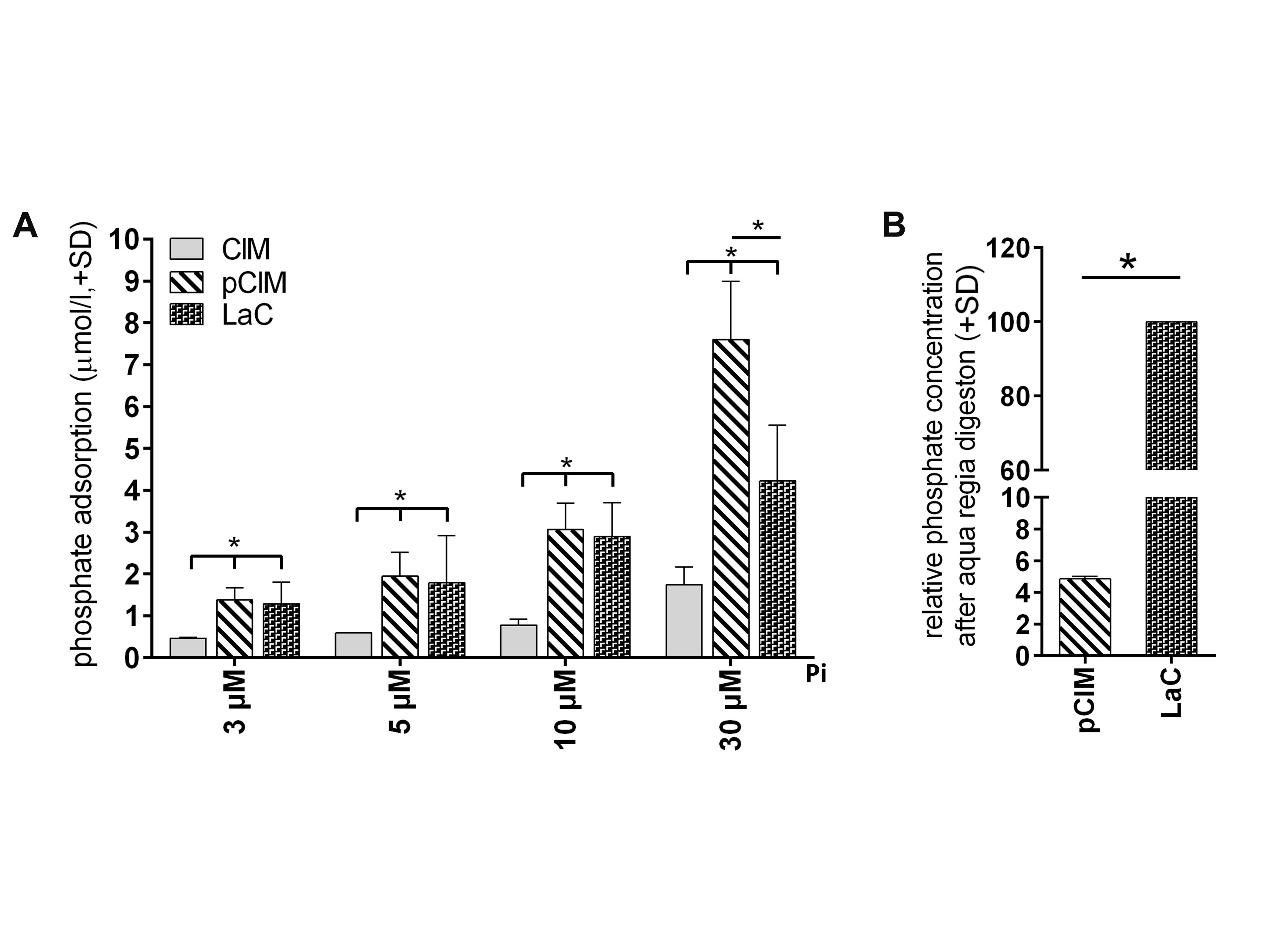

Supplement: Supplementary file 1 — Additional file 1. [file 12882_2022_2743_MOESM1_ESM.zip › Fig S1.JPG]

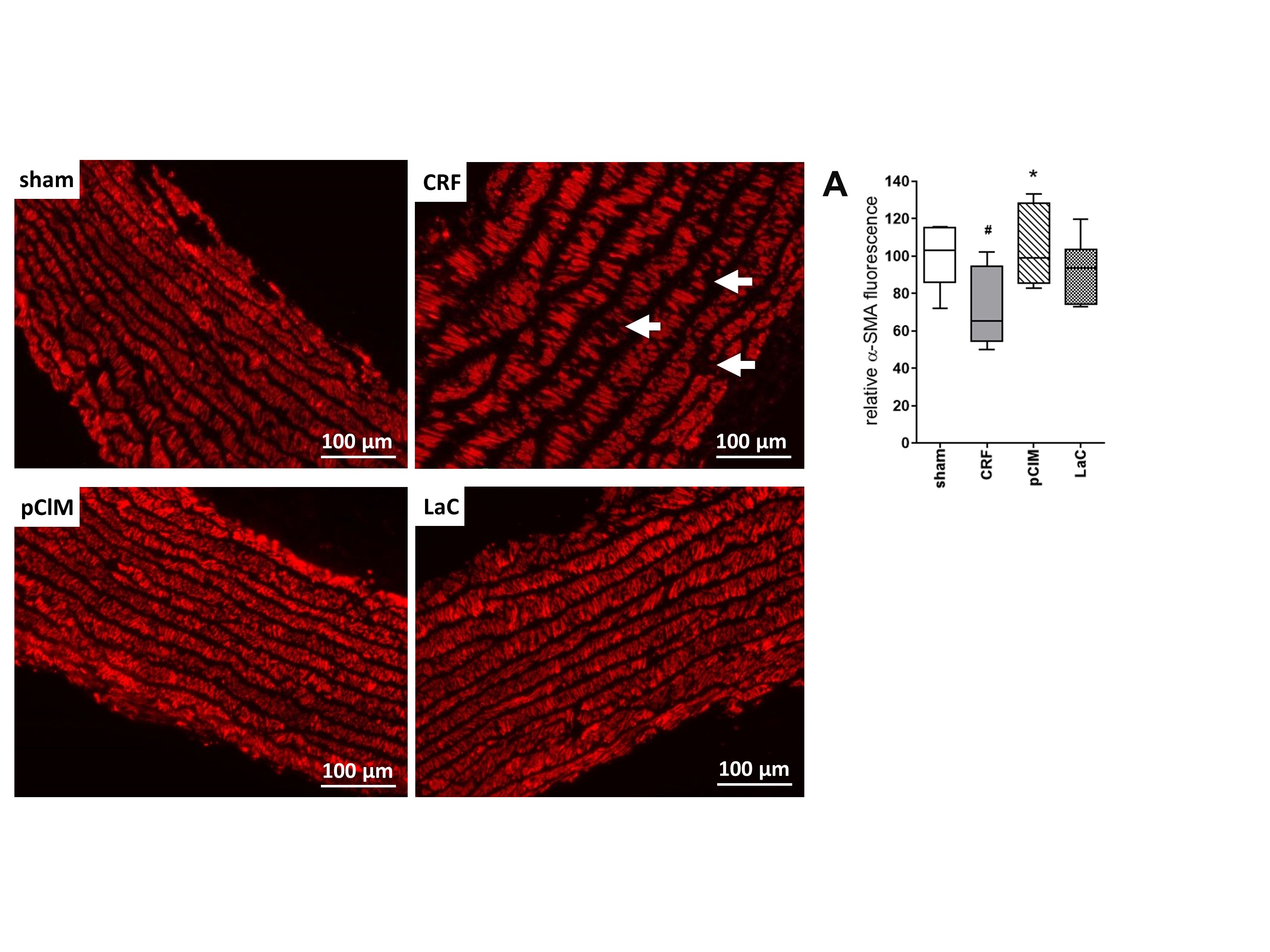

Supplement: Supplementary file 1 — Additional file 1. [file 12882_2022_2743_MOESM1_ESM.zip › Fig S2.jpg]

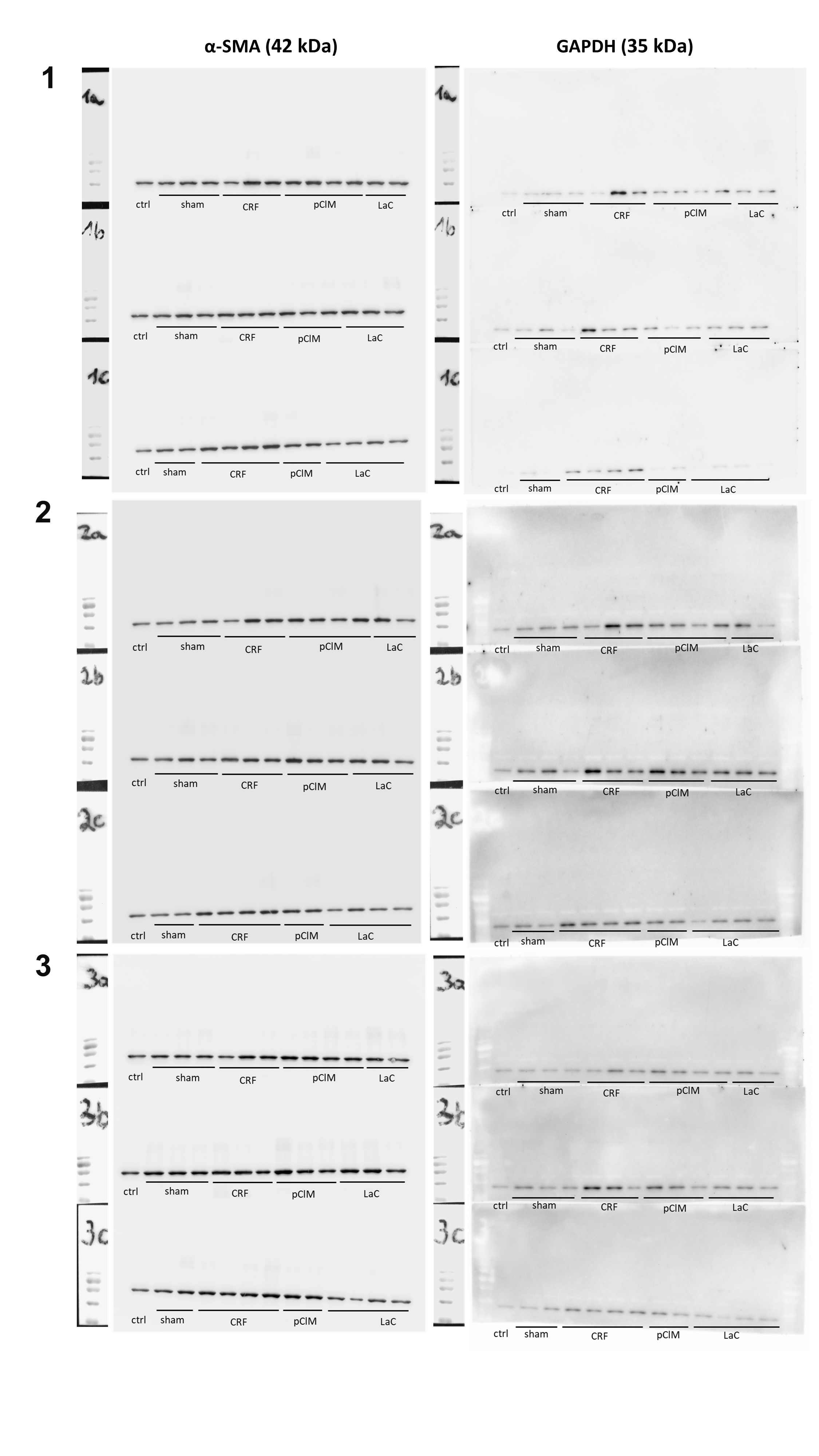

Supplement: Supplementary file 1 — Additional file 1. [file 12882_2022_2743_MOESM1_ESM.zip › Fig S3.jpg]

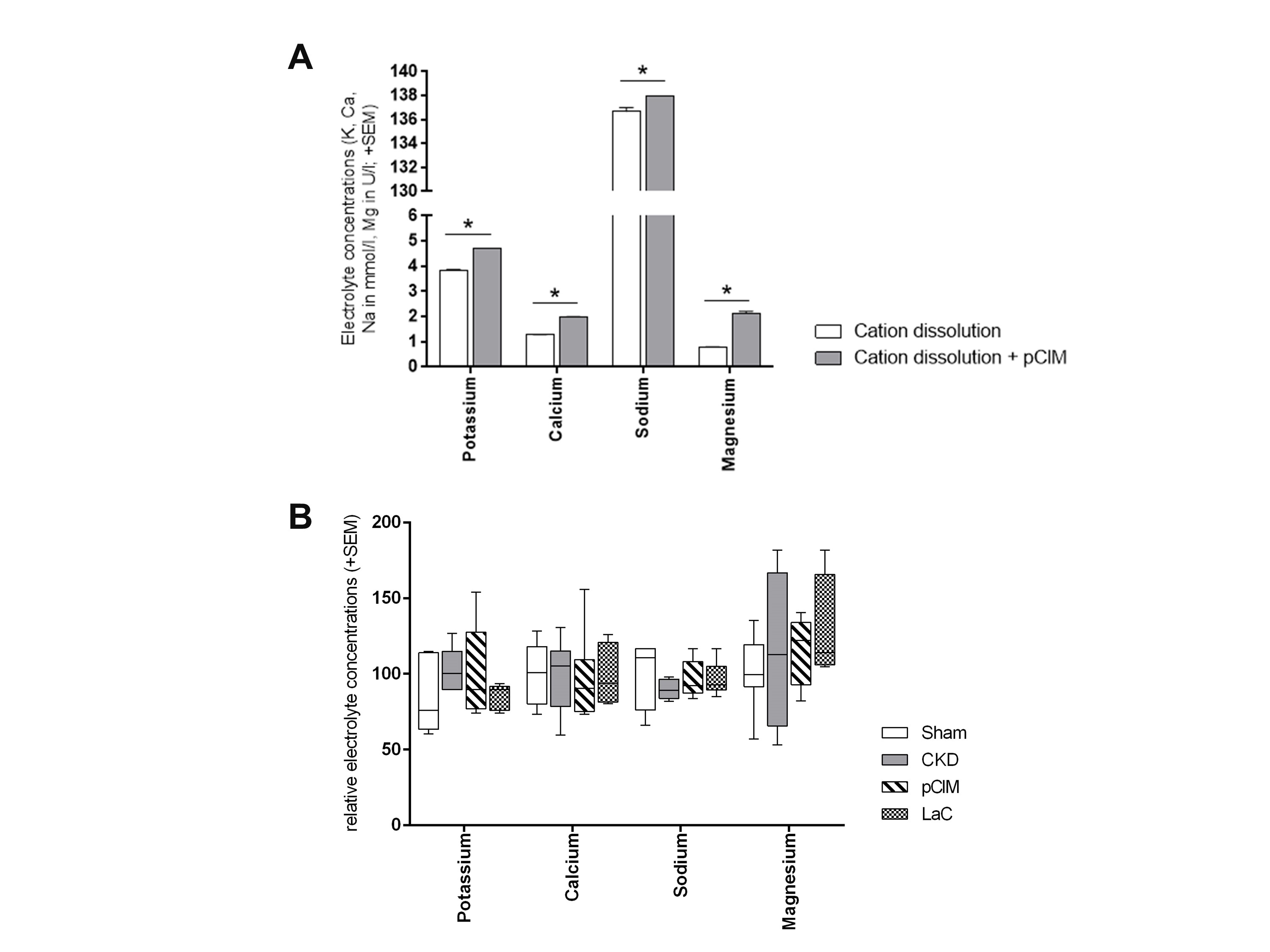

Supplement: Supplementary file 1 — Additional file 1. [file 12882_2022_2743_MOESM1_ESM.zip › Fig S4.jpg]
